# Possible Routes to Obtain Enhanced Magnetoresistance in a Driven Quantum Heterostructure with a Quasi-Periodic Spacer

**DOI:** 10.3390/mi12091021

**Published:** 2021-08-27

**Authors:** Arpita Koley, Santanu K. Maiti, Laura M. Pérez, Judith Helena Ojeda Silva, David Laroze

**Affiliations:** 1 Physics and Applied Mathematics Unit, Indian Statistical Institute, 203 Barrackpore Trunk Road, Kolkata 700 108, India; arpitakoley94@gmail.com; 2 Instituto de Alta Investigación, Universidad de Tarapacá, Casilla 7D, Arica 1000000, Chile; lperez@uta.cl (L.M.P.); dlarozen@uta.cl (D.L.); 3 Grupo de Física de Materiales, Universidad Pedagógica y Tecnológica de Colombia, Tunja 150003, Colombia; judith.ojeda@uptc.edu.co; 4 Laboratorio de Química Teórica y Computacional, Grupo de Investigación Química-Física Molecular y Modelamiento Computacional (QUIMOL), Facultad de Ciencias, Universidad Pedagógica y Tecnológica de Colombia, Tunja 150003, Colombia

**Keywords:** magnetoresistance, light irradiation, quasi-periodic spacer, tight binding framework, Floquet–Bloch ansatz, Green’s function formalism, quantum heterostructure

## Abstract

In this work, we perform a numerical study of magnetoresistance in a one-dimensional quantum heterostructure, where the change in electrical resistance is measured between parallel and antiparallel configurations of magnetic layers. This layered structure also incorporates a non-magnetic spacer, subjected to quasi-periodic potentials, which is centrally clamped between two ferromagnetic layers. The efficiency of the magnetoresistance is further tuned by injecting unpolarized light on top of the two sided magnetic layers. Modulating the characteristic properties of different layers, the value of magnetoresistance can be enhanced significantly. The site energies of the spacer is modified through the well-known Aubry–André and Harper (AAH) potential, and the hopping parameter of magnetic layers is renormalized due to light irradiation. We describe the Hamiltonian of the layered structure within a tight-binding (TB) framework and investigate the transport properties through this nanojunction following Green’s function formalism. The Floquet–Bloch (FB) anstaz within the minimal coupling scheme is introduced to incorporate the effect of light irradiation in TB Hamiltonian. Several interesting features of magnetotransport properties are represented considering the interplay between cosine modulated site energies of the central region and the hopping integral of the magnetic regions that are subjected to light irradiation. Finally, the effect of temperature on magnetoresistance is also investigated to make the model more realistic and suitable for device designing. Our analysis is purely a numerical one, and it leads to some fundamental prescriptions of obtaining enhanced magnetoresistance in multilayered systems.

## 1. Introduction

A magnetoresistance (MR) effect is defined as the change in electrical resistance of some conducting materials (preferably ferromagnets [1,2]) in response to an applied magnetic field [1,3,4,5,6,7,8,9,10]. There exist several kinds of magnetoresistance phenomena due to the variation in the structural and geometrical effects of different materials [11,12,13], the orientation of the applied magnetic field to them [12,13,14,15], and the presence of quantum mechanical interactions such as electron’s spin-orbit coupling [4,16,17,18,19,20,21,22], to name a few. Depending on these circumstances, magnetoresistance in multicomponent or multilayered systems can be categorized into four forms such as, giant magnetoresistance (GMR) [23,24,25,26,27,28,29,30], tunnel magnetoresistance (TMR) [31,32,33,34], colossal magnetoresistance (CMR) [35,36,37], and extraordinary magnetoresistance (EMR) [38,39,40].

In the present work, we are interested in the giant magnetoresistance (GMR) effect [41,42,43,44,45,46,47,48,49,50,51,51], which was undoubtedly one of the groundbreaking works in the late 80s. The Nobel winning work [6,9,23,26,29] in the year 2007 by Albert Fert and Peter Grünberg has enriched the area of spintronics along with immense technological progress such as magnetic recording, storage and sensor industries, and many more [52,53,54,55].

The phenomenon of magnetoresistance had set its journey through the weird property of nickel and iron in the presence of an external magnetic field, and Thomson measured the change of resistance for two specific directions of magnetization in the year 1856 [14]. Actually, spin-orbit coupling of electron is responsible for this anomalous observation and it is known as anisotropic magnetoresistance (AMR) [14,15]. After that, another notable work in this context was performed by Sir Nevill Francis Mott in early 1936 and he gave a qualitative explanation for the relatively high resistance in transition metals using the electron theory of metals [56]. High resistance occurs due to the greater probability of electron scattering between conducting ‘*s*’ states and unoccupied ‘*d*’ states. On the other hand, transition materials with unoccupied ‘*d*’ states show ferromagnetic or paramagnetic behavior. Thus, it was understood that there is a connection between magnetism and electrical conductivity. Gerlach and his co-workers extensively studied the resistance of nickel with respect to temperature and external magnetic field, and the result showed that in the neighborhood of the Curie temperature, resistance becomes less in the presence of a $\vec{B}$ field.

To explain that fact, Mott considered the spin of the electrons in the conduction states due to the presence of an external magnetic field. In lower temperature, as soon as spontaneous magnetization reaches its highest value, the spin direction of unoccupied ‘*d*’ states becomes antiparallel to the applied $\vec{B}$ field, resulting only in one kind of spin transition between the ‘*s*’ and ‘*d*’ states. However, above the Curie temperature, holes of the ‘*d*’ state also split into two spin channels, and therefore, both kinds of spin transitions are possible. Consequently, it increases the resistance of transition elements possessing partially filled ‘*d*’ states. Following the band structure of ferromagnetic metals, it reveals that the density of states as well as the scattering rates for up and down spin electrons are not equal at the Fermi energy. Thus, resistivities for different spin channels become separated and give rise to the phenomenon of magnetoresistance, depending on the magnetization direction.

At the end of the 1970s, the fabrication of a few nanometer thick multilayers became possible using several new techniques of epitaxial growth [5,34,57,58]. Experimentally, it was found that the presence of a non-magnetic (NM) spacer having a few atomic layer thick (less than a mean free path of conduction electrons to maintain the GMR effect), sandwiched between ferromagnetic multilayers under applied magnetic field, caused a significant reduction in electrical resistance [23,29,57,58,59]. The work Grünberg et al. [6,29] was associated with a trilayer structure of Fe/Cr/Fe, whereas the discovery of Fert et al. [9,26] belonged to several layers (60) of Fe/Cr. The value of GMR decreased by almost 50% in the latter case as it was performed at the temperature 4.2 K, whereas the other one achieved 10% at room temperature, with three Fe layers separated by two Cr layers.

**Figure 1:**
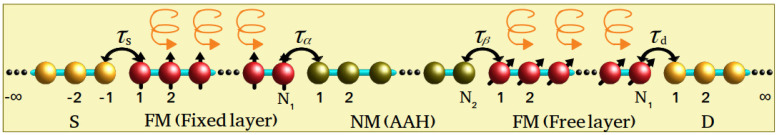
(Color Online). Schematic diagram of the magnetoresistance setup in a trilayer structure, which is connected to a non-magnetic source (S) and drain (D) electrodes through the couplings $\tau_{S}$ and $\tau_{D}$, respectively. The bridging conductor is made up of a non-magnetic spacer ($N_{2}$ sites), subjected to AAH potential, and two ferromagnetic layers ($N_{1}$ sites in each layer) that are irradiated with light.

In our work, we consider a one-dimensional trilayer structure (see Figure 1), where a NM spacer with quasi-periodic site energies is clamped between two ferromagnetic layers. The magnetization of one magnetic layer is *fixed* along +Z direction, while for the other ferromagnetic layer, the magnetization direction is *free*, and depending on its magnetic moment orientation [8,9,23,48], resistance changes accordingly. It is straightforward to understand that, if the magnetization of the free layer is kept parallel to the fixed one, resistance becomes very low due to low scattering rates, and an antiparallel orientation suggests a high scattering rate, yielding a large resistance. Now, this spin-dependent transport [32,55,60,61] behavior can be tuned externally by modifying the characteristics of NM spacer and ferromagnetic layers. The site energies of the NM spacer is altered by cosine modulation following the AAH form [62,63,64,65,66,67,68,69,70,71,72,73], whereas the hopping parameter in ferromagnetic chains is rearranged by Floquet–Bloch anstaz [60,74,75,76,77,78,79] within a minimal coupling scheme due to light irradiation on them. The Hamiltonian of a layered nanojunction is constructed with the TB approximation [31,67] including only the contribution from the nearest-neighbor hopping (NNH) integral. Interplay between the fractal-like gapped transmission spectrum [68,69] because of the AAH modulation and the renormalized hopping due to light irradiation [74,75] may lead to several interesting features in the GMR effect. Our present analysis would be beneficial in developing a deeper insight into the role of correlated disorder and light irradiation in the context of a magnetoresistance study.

The rest of our work is arranged as follows. In Section 2, we present the GMR setup and the theoretical prescription for the calculations. All of the results are presented and thoroughly investigated in Section 3, and finally, we conclude our important findings in Section 4.

## 2. Magnetoresistance Setup and Theoretical Framework

### 2.1. Quantum Heterostructure and the TB Hamiltonian

The schematic diagram of a trilayer structure between a one-dimensional semi-infinite non-magnetic source and a drain is depicted in Figure 1, where the left ferromagnetic layer is assumed to be a fixed layer with a magnetic moment in each site oriented along the +Z direction, the middle layer is the non-magnetic spacer, and the last one is another ferromagnetic layer in which the magnetization direction can be altered selectively. The properties of each such layer can be modified and the combined effect is discussed in our work. Now, for the magnetoresistance measurement, the magnetic moments of electrons’ spin in the free layer are chosen to be parallel and antiparallel with respect to the fixed layer [6,9,12]. The whole arrangement of the nanojunction is represented by a tight-binding framework considering the NNH integrals.

The Hamiltonian of the magnetic–non-magnetic–magnetic (M–NM–M) quantum heterostructure is divided into different sub-Hamiltonians, associated with different parts of the junction, and it is can be written as [31,48]

(1) $$H=H_{S}+H_{con}+H_{D}+H_{tun}$$

where $H_{S}$ and $H_{D}$ denote the Hamiltonians for the source and drain electrodes, respectively, and $H_{con}$ represents the bridging conductor part and the last one, i.e., $H_{tun}$ includes the tunneling junctions between electrodes and magnetic layers.

We start with the conducting channel Hamiltonian $H_{con}$ that can be further divided into three sections corresponding to ferromagnetic layers, a NM spacer, and coupling between the junctions. Ferromagnetic layers, each having $N_{1}$ number of lattice sites, on both sides of the spacer subjected to light irradiation are expressed in the TB form as

(2) $$H_{M}=\sum_{n=1}^{N_{1}}c_{n}^{†}\left(\epsilon_{n}-{\vec{h}}_{n}.\vec{\sigma }\right)c_{n}+\sum_{n=1}^{N_{1}-1}\left(c_{n}^{†}\tilde{t}c_{n+1}+h.c.\right).$$

Each term of this equation is explicitly described here. $c_{n}^{†}\left(c_{n}\right)$ is the fermionic creation (annihilation) operator for the *n*th site and their components obey anticommutation relation. The first term of Equation (2) denotes the on-site energy of magnetic chains in the presence of a spin-flip scattering mechanism [18,60,61] between the magnetic moments of the *n*th site and the incoming electron, and consequently, the potential profiles of the electrons belonging to two spin states become separated. $\epsilon_{n}$ is the potential energy at the *n*th site without any kind of interaction term. ${\vec{h}}_{n}$ is the spin-flip scattering vector, and its orientation is described in the usual spherical polar co-ordinate system with polar angle $\theta_{n}$ and azimuthal angle $\varphi_{n}$. $\vec{\sigma }$ is the conventional Pauli spin vector. The second term represents the renormalized nearest-neighbor hopping integral $\tilde{t}$ due to the perturbation of light irradiation [74,75]. Here, all of the terms are expressed in the following forms:

$$c_{n}=\left(\begin{array}{c} c_{n↑}\\ c_{n↓} \end{array}\right);c_{n}^{†}=\left(\begin{array}{cc} c_{n↑}^{†} & c_{n↓}^{†} \end{array}\right);\epsilon_{n}=\left(\begin{array}{cc} \epsilon_{n↑} & 0\\ 0 & \epsilon_{n↓} \end{array}\right);$$

$${\vec{h}}_{n}.\vec{\sigma }=\left(\begin{array}{cc} h_{n}\cos\theta_{n} & h_{n}\sin\theta_{n}e^{-i\varphi_{n}}\\ h_{n}\sin\theta_{n}e^{i\varphi_{n}} & -h_{n}\cos\theta_{n} \end{array}\right);\tilde{t}=\left(\begin{array}{cc} \tilde{t} & 0\\ 0 & \tilde{t} \end{array}\right).$$

The renormalized hopping strength $\tilde{t}$ is obtained from the following integration procedure [74,75]:

(3) $$\begin{array}{ccc} \tilde{t} & = & \frac{t}{T}\int_{0}^{T}e^{j(p-q)\Omega \tau }e^{\vec{A}.\vec{a}}d\tau \\ & & =tJ_{\left(p-q\right)}\left(\Lambda \right) \end{array}$$

where *t* denotes the hopping strength in the absence of light irradiation. $J_{\left(p-q\right)}$ is the (p−q)th order Bessel function of the first kind, and its argument Λ simplifies to $A_{x}a$ (*a* being the lattice spacing) as per our consideration of one-dimensional layered geometry. $A_{x}$ is the amplitude of vector potential $\vec{A}\left(\tau \right)$ that enters this calculation through the Floquet–Bloch (FB) ansatz within the minimal coupling scheme to incorporate the effect of light irradiation into the TB Hamiltonian. The general form of $\vec{A}\left(\tau \right)$ reads as [74,75]

$$\vec{A}\left(\tau \right)=\left\{A_{x}\sin\left(\Omega \tau \right),A_{y}\sin\left(\Omega \tau +\phi \right)\right\},$$

where $A_{x}$ and $A_{y}$ are the amplitudes, and ϕ is the phase. Tuning these parameters, the light is made to be linearly, circularly, or elliptically polarized. For our one-dimensional nanojunction, $A_{y}$ and ϕ do not contribute, and the problem becomes quite simpler. Ω is the frequency of the driving electromagnetic field, and the time period T=2π/Ω. $\vec{A}\left(\tau \right)$ obeys the relation $\vec{A}\left(T+\tau \right)=\vec{A}\left(\tau \right)$. For a detailed description of FB theory, see References [19,74,75,76,77,78,79].

The TB Hamiltonian of the NM spacer, possessing $N_{2}$ lattice sites, subjected to AAH site potentials obtains the form [62,63,65]

(4) $$H_{NM}=\sum_{n=1}^{N_{2}}c_{n}^{†}\left(w\cos\left(2\pi nb+\phi_{\nu }\right)\right)c_{n}+\sum_{n=1}^{N_{2}-1}\left(c_{n}^{†}{\mathrm{tc}}_{n+1}+h.c.\right)$$

where *w* is the strength of the cosine modulation, $\phi_{\nu }$ is the AAH phase factor, and *b* is an irrational number. In our calculation, we chose $b=(1+\sqrt{5})/2$ (golden number) to make the potential incommensurate. This typical value of *b* is mostly used in the literature, though any other irrational number can also be taken into account and the physics remains unchanged. Since the spacer is an NM one, the on-site energy becomes spin independent, and thus, $\epsilon_{n↑}=\epsilon_{n↓}$.

For the side-attached 1D electrodes, those are assumed to be semi-infinite and non-magnetic in nature, the Hamiltonians read as

(5) $$H_{S}=H_{D}=\sum_{n}{b_{n}}^{†}\epsilon_{0}b_{n}+\sum_{n}\left(b_{n}^{†}t_{0}b_{n+1}+h.c.\right)$$

The site index *n* in the source electrode runs from −1 to −∞, while for the drain, it varies from 1 to ∞. $\epsilon_{0}$ and $t_{0}$ are the on-site energy and NNH strength, respectively. $b_{n}$ is the anticommutating fermionic operator similar to $c_{n}$, as used in the conductor.

Finally, the tunneling Hamiltonian $H_{tun}$ that connects source (*S*) and drain (*D*) electrodes to ferromagnetic chains can be written as

(6) $$H_{tun}={b_{-1}}^{†}\tau_{s}c_{1}+b_{1}^{†}\tau_{d}c_{N_{1}}+h.c.$$

where $\tau_{s}$ and $\tau_{d}$ represent the coupling strengths between the source end to the first lattice site of the fixed magnetic layer and the first lattice point of the drain electrode to the end site of the free magnetic layer, respectively (see Figure 1).

### 2.2. Theoretical Framework

The determination of magnetoresistance requires the conductances in parallel (P) and antiparallel (AP) configurations of magnetic moments to be between the fixed and free magnetic layers. The conductance can be evaluated from the transmission probabilities of up and down spin electrons following the Landauer definition, whereas spin-dependent transmission probabilities are computed with the help of the well known non-equilibrium Green’s function (NEGF) technique [80]. In the NEGF approach, the effects of the side attached electrodes are incorporated through finite dimensional self-energy matrices, and the effective Green’s functions are [80,81,82,83,84]

(7) $$G^{r}={\left(G^{a}\right)}^{†}={\left(EI-H_{con}-\sum_{S}-\sum_{D}\right)}^{-1}$$

where *E* is the energy of an incident electron and *I* represents the identity matrix. All of the matrices in Equation (7) are of the dimension $2\left(2N_{1}+N_{2}\right)\times 2\left(2N_{1}+N_{2}\right)$. $\sum_{S}$ and $\sum_{D}$ are self-energy matrices of source and drain electrodes, respectively. Using the above retarded and advanced Green’s functions, we determine the spin-dependent transmission co-efficients $T_{\sigma \sigma^{\prime }}$ from the Fisher–Lee relation [85]

(8) $$T_{\sigma \sigma^{\prime }}=\mathrm{Tr}\left[\Gamma_{S}^{\sigma }G^{r}\Gamma_{D}^{\sigma^{\prime }}G^{a}\right]$$

where the coupling matrices $\Gamma_{S}^{\sigma }$ and $\Gamma_{D}^{\sigma^{\prime }}$ are

$$\Gamma_{S(D)}^{\sigma (\sigma^{\prime })}=i\left[\sum_{S(D)}^{\sigma (\sigma^{\prime })}-{\left(\sum_{S(D)}^{\sigma (\sigma^{\prime })}\right)}^{†}\right].$$

Computing the individual spin-dependent transmission components, we obtain the net up and down spin transmission probabilities as follows: $T_{↑}=T_{↑↑}+T_{↓↑}$ and $T_{↓}=T_{↓↓}+T_{↑↓}$.

Using the Landauer integral, conductance $G_{\sigma \sigma^{\prime }}$ is written as [80]

(9) $$G_{\sigma \sigma^{\prime }}=\left(e^{2}/h\right)L_{0}^{\sigma \sigma^{\prime }}$$

where

$$L_{0}^{\sigma \sigma^{\prime }}=-\int T_{\sigma \sigma^{\prime }}\left(E\right)\frac{\partial f}{\partial E}dE.$$

e,h are electronic charge and Planck’s constant, respectively, and *f* is the Fermi–Dirac distribution function. At absolute zero temperature, Equation (9) boils down to $G_{\sigma \sigma^{\prime }}=\left(e^{2}/h\right)T_{\sigma \sigma^{\prime }}$.

Finally, we define GMR through the relation [6,9]

(10) $$GMR=\left|\frac{G_{P}-G_{AP}}{G_{P}+G_{AP}}\right|$$

where $G_{P}$ and $G_{AP}$ include ‘net’ electron transfer (both up and down) for the parallel and anti-parallel cases, respectively. The definition of GMR suggests that its value can vary from zero to one hundred percent, and our ultimate goal is to achieve the highest value of GMR.

## 3. Numerical Results and Discussion

In what follows, we present our numerical results, which include the spin-dependent transmission probabilities through the nanojunction, energy band diagrams of the heterostructure sandwiched between the contact electrodes, and GMR characteristics under different input conditions.

Before presenting the results, let us briefly mention the physical parameter values that are kept constant throughout the numerical calculations. For the electrodes, we set $\epsilon_{0}=0$ and $t_{0}=2\;$ eV. The site energies in the ferromagnetic layers are taken as $\epsilon_{n↑}=\epsilon_{n↓}=0$ and the NNH strength t=1 eV. For the NM spacer, the cosine modulation strength *w* is fixed at 1 eV. The strengths of the magnetic moments are considered uniform (viz, $h_{n}=h$), and we set h=1 eV. For the fixed magnetic layer, the moments are orientated along the +Z direction, i.e, $\theta_{n}=0$. In contrast, for the free layer, $\theta_{n}$ becomes zero or π depending on whether the moments are oriented along the +Z (parallel configuration) or −Z (anti-parallel configuration) directions. The coupling strengths between different layers of the heterostructure as well as the coupling between electrodes and magnetic layers are fixed at 1 eV. The lattice sites are $N_{1}=10$ and $N_{2}=20$. The energies are measured in units of electron-volt (eV), and unless stated otherwise, we fix the system temperature to absolute zero.

In studying the effect of light irradiation, we restrict ourselves in the *high-frequency* limit, defined as [74,75] ħΩ>>t. There are two fundamental reasons for choosing this limiting condition. First, the Floquet bands are almost decoupled to each other, and thus, only the lowest order band contributes, i.e., p=q=0 [19,60,61]. Second, it is well-known that a D-dimensional driven quantum system is equivalent to a D+1-dimensional undriven quantum system. Thus, we need to consider several identical copies of the parent lattice sites, which unnecessarily increase the system size, and eventually the system size will be much larger than the spin diffusion length (SDL). Under this situation, the spin-dependent transport phenomena and the GMR effect are no longer visible. The average spin diffusion length of the usable ferromagnetic materials is of the order of 50 nm [59], which suggests that we can safely consider almost 500 lattice sites (assuming the lattice spacing ∼1).

In order to satisfy the condition of a high-frequency limit of the incident light, Ω should be of the order of $10^{15}\;$ Hz or more. It corresponds to the regime of near-ultraviolet/extreme ultraviolet. The light intensity is ∼$10^{5}\;$ W/m ${}^{2}$, and it is far below the upper limit of experimentally realizable intensities [86,87]. The chosen intensity does not damage the physical system. For this light irradiation, the electric field E becomes ∼$10^{4}\;$ V/m, while the magnetic field *B* is ∼$10^{-5}\;$ T. Since the associated magnetic field *B* is too weak, its effect is neglected.

### 3.1. Spin-Dependent Transmission Probabilities and GMR

**Figure 2:**
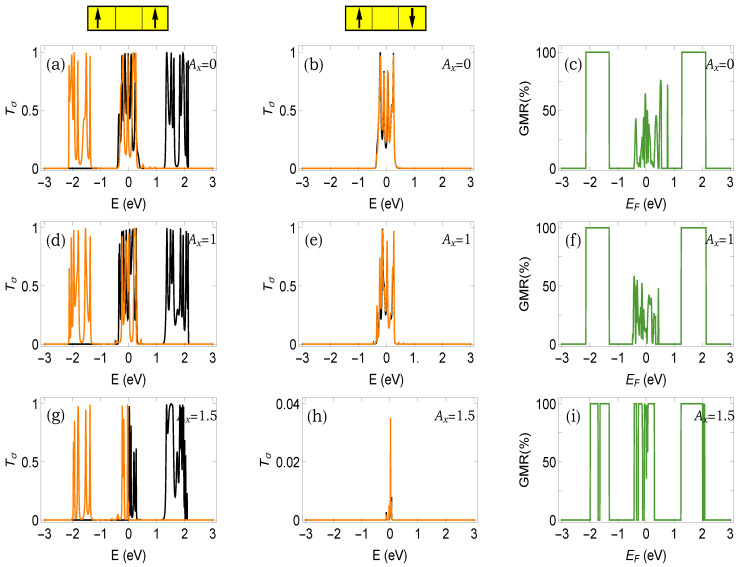
(**a**–**i**) (Color Online). Transmission probabilities $T_{\sigma }$ as a function of energy (first and second columns, $T_{↑}\to$ orange, $T_{↓}\to$ black) and GMR as a function of Fermi energy (last column) under three typical values of light parameter $A_{x}$. AAH phase $\phi_{\nu }=0$.

We start presenting our results with Figure 2, where the net up and down spin transmission probabilities along with the GMR are shown under three different input conditions of the light parameter. The effects of cosine modulation, light irradiation, and the interplay between them bring several interesting new features that we discuss here one by one. For the parallel configuration, the transmission peaks are merged into two broad energy regions followed by a zero transmission gap. On the other hand, for the anti-parallel configuration, only one energy window, across zero energy, is available where finite transmission occurs. For each such transmission window, several peaks and dips are obtained. All of these features are associated with the fractal like energy bands and gaps of the AAH spacer. Even though the AAH spacer provides *three* distinct energy bands followed by two gaps, in the transmission spectrum, we obtain less transmission zones where finite transmission takes place. The appearance of two transmission windows for the parallel case and a single transmission window for the anti-parallel case solely depends on the common energy channel of the three-layered heterostructure (viz the M–NM–M spacer). Once the arrangement of magnetization becomes altered from the parallel to the anti-parallel configuration, the common energy window becomes modified, and following the common energy region, we obtain the net transfer of electrons across the junction. A more clear picture of this mechanism will be evident from our forthcoming analysis of the energy band diagram.

The shifting of the up and down spin transmission peaks in each $T_{\sigma }$-*E* spectrum is due to the spin-dependent scattering of electrons with local magnetic moments in the ferromagnetic layers. In the presence of this scattering, the effective site energies of up and down spin electrons are different from each other, resulting in a finite mismatch between the energy eigenvalues and, thus, the transmission spectra. This phenomenon of channel mismatch in the presence of spin-dependent scattering is well known in the literature and has been studied in different contexts considering simple and complex magnetic systems. However, for a heterostructure, where one part is subjected to quasi-periodic modulation, several additional signature are found, and here, we provide such an example by investigating the magnetoresistance phenomenon.

More appealing features are obtained when we include the effect of light irradiation. In presence of light, the NNH strength becomes renormalized following the relation given in Equation (3), and therefore, the energy band spectrum is modified, which directly affects the transmission probabilities. Comparing the transmission spectra shown in Figure 2, it is clearly seen that the band narrowing occurs with enhancing the amplitude $A_{x}$, and for the AP configuration, it becomes more prominent. We can reach a situation where finite transmission is obtained for the parallel configuration, whereas an almost vanishing transmission occurs for the anti-parallel configuration. Under this condition, a high degree of GMR is naturally expected. The entire mechanism of obtaining finite transmission across the heterostructure depends on the availability of the overlap energy channels, and that can be fully controlled by the modulated site energies and the renormalized hopping integrals.

From the spin-dependent transmission probabilities in parallel and anti-parallel configurations (first and second columns of Figure 2), the nature of GMR spectra (third column of Figure 2) can be easily understood. In the absence of irradiation, a large degree of magnetoresistance is of course obtained, but mostly towards the energy band edges, which is not quite favorable from the experimental point of view since placing the Fermi energy near the band edge is quite difficult. However, even for this case, moderate GMR is obtained near the band center, providing peaks and dips, and these aspects are solely due to the existence of the AAH spacer. With increasing strength of $A_{x}$, we can achieve a higher GMR near the band center and finally reach a situation where almost a hundred percent GMR is available. Achieving a high degree of GMR (of course, one hundred percent is the best option) near the band center is the primary goal of our analysis, and we hope that we approach that limit.

### 3.2. Energy Band Diagram of the Quantum Heterostructure

To understand the precise role of the AAH spacer along with the effect of light irradiation, it is indeed required to analyze the energy band spectra of the individual layers, since the net electron transfer through a layered structure depends on the energy channels of all of these layers.

**Figure 3:**
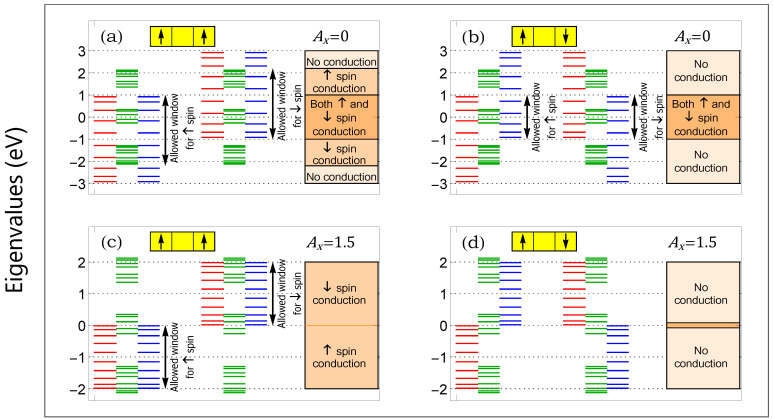
(**a**–**d**) (Color Online). Energy levels of three different layers of the heterostructure, where the red, green, and blue energy bands are associated with the free layer, the NM spacer, and the fixed layer, respectively. Two different cases, irradiation-free and with irradiation, are shown. In each of the spectra, the left three colored bands are for the up spin electrons, while the other three are for the down spin electrons. On the right most side, the uniformly filled colored regions are used to illustrate through which energy zone an electron can transfer or not. The AAH phase $\phi_{\nu }$ is fixed to zero.

In Figure 3, we display the energy band diagrams of three different layers both for the parallel and anti-parallel configurations. In each spectrum, two different sets of three colored energy bands are shown. The left one corresponds to the energy levels for the up spin electrons, while the right one is associated with the down spin electrons. For better viewing of the distinct energy levels, we draw a single horizontal line in each of the eigenvalues.

The positions of up (red) and down (blue) spin bands in the ferromagnetic layers are associated with the effective site energies in the presence of the spin-dependent scattering. However, as the spacer is non-magnetic, the green window becomes unaltered for both the parallel and anti-parallel arrangements of the magnetic moments in the two magnetic layers. The transfer of electron through the junction occurs only through the common energy channels of all three layers. Therefore, adjusting the available energy channels either of the AAH spacer or the magnetic layers, or of both, we can regulate the electron transfer selectively. This is the key advantage of considering a layer structure over a single layer.

In the absence of light, we find that, for the parallel configuration, both electrons can pass through the center of the band while only up or down can propagate across this window (Figure 3a). Once the moments in the free layer are oriented along the −Z direction, only the central energy region allows electrons to pass through the junction (Figure 3b). As a result of this, we obtain a large GMR (almost a hundred percent) across the two edges of the energy band. The finite but reasonably smaller value of GMR near the band center is obtained due to the conductance mismatch between the two configurations. The lowering of a mismatch yields a reduction in the GMR value, as expected. Suitably irradiating the ferromagnetic layers, we can find a situation where almost perfect blocking of an electron takes place in the anti-parallel configuration, and thus, even very close to the energy band center, we obtain a large GMR.

### 3.3. Tuning of GMR

In this subsection, we explore the possible tuning mechanisms of GMR by regulating the physical parameters in a wide range for the sake of completeness of our analysis.

**Figure 4:**
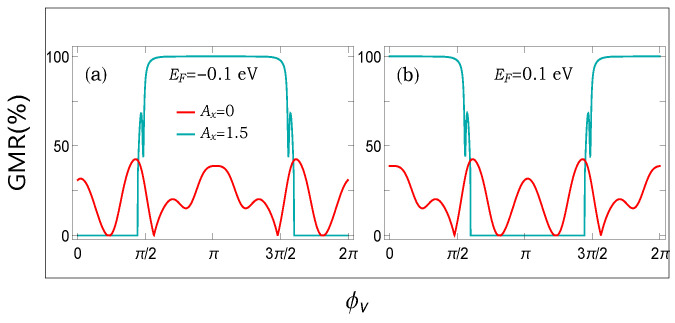
(**a**,**b**) (Color Online). Variation in GMR with the AAH phase $\phi_{\nu }$ at two different Fermi energies both in the absence and presence of light irradiation.

From the above discussion, it is already established that the spacer has an important role in magnetoresistance. Thus, if we can tune the energy band diagram of it, then definitely, we can have some variations in GMR. To substantiate this issue, in Figure 4, we show the dependence of GMR on the AAH phase factor $\phi_{\nu }$ for the two typical Fermi energies. The results are computed at two distinct values of the light parameter $A_{x}$. A wide variation with $\phi_{\nu }$ is obtained. This is solely due to the modification of the energy levels of the spacer. Another important point is that the selection of Fermi energy is also crucial to obtain a large GMR for a particular set of other physical parameters. Thus, to obtain a most favorable response, a thorough scan over the wide range of the parameters is undoubtedly required.

**Figure 5:**
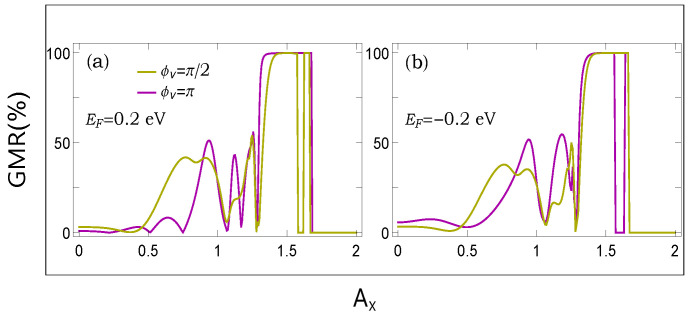
(**a**,**b**) (Color Online). Dependence of GMR with $A_{x}$ at two typical Fermi energies. The results are shown for two different AAH phases.

In the same footing, in Figure 5, we present the dependence of GMR with light parameter $A_{x}$ at two typical Fermi energies. The results are computed for two distinct AAH phase factors. For lower values of $A_{x}$, there are some oscillations, and for higher values of $A_{x}$, GMR reaches its maximum limiting value. The underlying physical mechanism relies on the renormalization of the effective hopping of the ferromagnetic layers and the interplay between the available energy channels of these magnetic layers along with the spacer.

**Figure 6:**
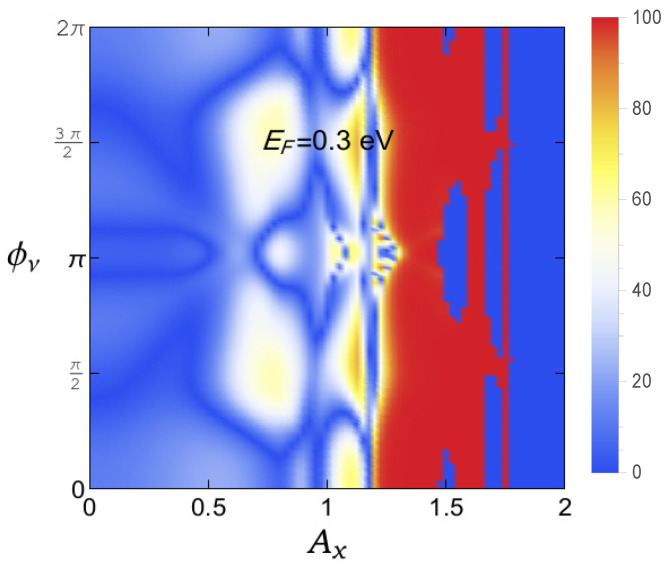
(Color online). Simultaneous variation (density plot) of GMR as functions of $A_{x}$ and AAH phase $\phi_{\nu }$. Here, we chose E=0.3 eV.

From the results presented in both Figure 4 and Figure 5, we see that there are finite possibilities to control magnetoresistance by tuning either the AAH phase or the light parameter, or both. Now, in order to inspect the dependence of GMR, when we simultaneously vary both these two factors viz AAH phase and the light parameter, in Figure 6, we show a density plot of GMR with the quantities, setting the Fermi energy at $E_{F}=0.3\;$ eV.

The first impression is that we can vary the degree of magnetoresistance in a reasonable range, ranging from a very low one to the maximum limiting value (near to cent percent). Having tunable devices based on the magnetoresistance phenomena is undoubtedly helpful. The other crucial point is that any particular value of GMR persists over a broad range of physical parameters, which gives us confidence to verify our results with a suitable laboratory setup.

### 3.4. Effect of Temperature

**Figure 7:**
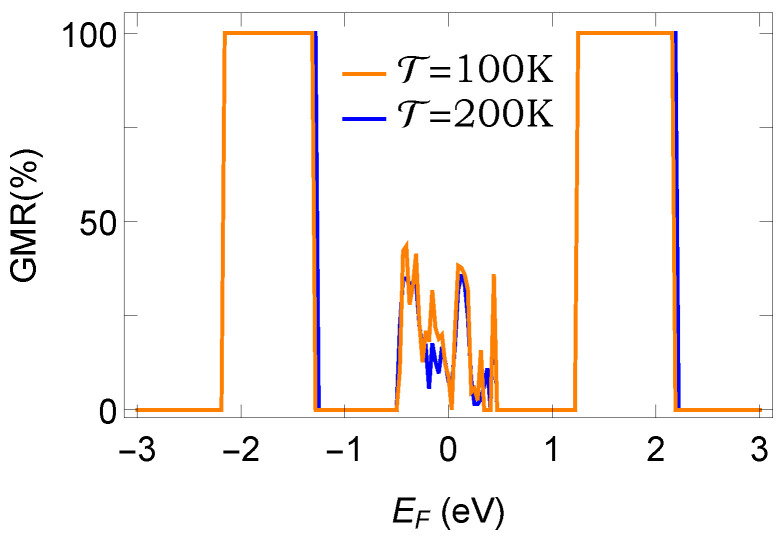
(Color Online). Temperature dependence: GMR as a function of Fermi energy at two different temperatures. Here, we consider $A_{x}=1$ and the AAH phase $\phi_{\nu }=0$.

The results analyzed so far are worked out at absolute zero temperature. Keeping in mind the possible experimental realization, finally we investigated the role of temperature on GMR. The results are presented in Figure 7, where we show the variation of GMR with Fermi energy $E_{F}$ at two distinct temperatures. Two curves almost overlap with each other for the entire energy window, which suggests that a favorable response can be obtained even in the large temperature limit. The reason is that the average energy level spacing is quite large due to small-scale systems compared with the thermal energy. Of course, when the temperature is too large (>400 K), the degree of GMR is quite less, which we confirm through our exhaustive numerics, but we do not need to go into that high temperature limit. Here, it is relevant to note that the range of temperatures is directly linked to the system size. We need to restrict the temperature in such a way that the thermal energy is always less than the average energy level spacing of the system. Along with this, the dimension of the system is also restricted in another way by considering the limiting value of the spin diffusion length (∼50 nm). If the dimension of the functional element is higher than the SDL, then it is hard to obtain a favorable response.

## 4. Closing Remarks

To conclude, in this work, we investigated for the first time, to the best of our knowledge, the interplay between cosine modulations and light irradiation on a magnetoresistive study within a tight-binding framework. We considered a one-dimensional quantum heterostructure where a non-magnetic spacer is clamped between two ferromagnetic layers. Among these two, the magnetization direction in one magnetic layer remains fixed, while for the other layer, it is free to change. The site energies of the NM spacer are modulated in the well-known AAH form, and the two magnetic layers are subjected to light irradiation. We essentially tried to develop the possible routes of engineering magnetoresistance considering the combined effects of AAH potential and light irradiation.

Following the Floquet–Bloch approach, we incorporated the effect of light within a minimal coupling scheme and computed the spin-dependent transmission probabilities for the determination of GMR with the help of Green’s function formalism. All of the results have been thoroughly discussed and explain the underlying mechanisms following the energy band diagrams of different segments of the heterostructure. Several interesting and important features have been emerged, as follows:

- A high degree of GMR was obtained; sometimes, it even reaches a hundred percent.
- The favorable response of GMR can be achieved even near the band center for the driven heterostructure, which is no longer available for the irradiation-free case.
- We can selectively tune GMR by means of either the AAH phase or the irradiation or by regulating both of these factors.
- The results are valid for a reasonable range of physical parameters. Moreover, the high degree of GMR still persists even for a large temperature limit.

Before ending this paper, we note that our analysis is not restricted to a specific physical system or material. It can be generalized to any simple and complex driven M–NM–M quantum heterostructure in the presence of such a quasi-periodic modulation. Different kinds of magnetic systems have already been used so far in the vast literature, and any such magnetic system is recommended. The crucial point of our analysis is the inclusion of an aperiodic NM spacer between the two magnetic layers and the application of light irradiation. These two factors play a central role in our analysis.

## References

[1] Zhang X., Li B.-Z., Sun G., Pu F.-C. (1997). Spin-polarized tunneling and magnetoresistance in ferromagnet/insulator(semiconductor) single and double tunnel junctions subjected to an electric field. Phys. Rev. B.

[2] Sarma S.D., Hwang E.H., Kaminski A. (2003). How to make semiconductors ferromagnetic: A first course on spintronics. Solid State Commun..

[3] He P., Hsu C.-H., Shi S., Cai K., Wang J., Wang Q., Eda G., Lin H., Pereira V.M., Yang H. (2019). Nonlinear magnetotransport shaped by Fermi surface topology and convexity. Nat. Commun..

[4] Jiang J., Tang F., Pan X.C., Liu H.M., Niu X.H., Wang Y.X., Xu D.F., Yang H.F., Xie B.P., Song F.Q. (2015). Signature of Strong Spin-Orbital Coupling in the Large Nonsaturating Magnetoresistance Material *W**T**e*_2_. Phys. Rev. Lett..

[5] Moodera J.S., Kinder L.R., Wong T.M., Meservey R. (1995). Large Magnetoresistance at Room Temperature in Ferromagnetic Thin Film Tunnel Junctions. Phys. Rev. Lett..

[6] Binasch G., Grünberg P., Saurenbach F., Zinn W. (1989). Enhanced magnetoresistance in layered magnetic structures with antiferromagnetic interlayer exchange. Phys. Rev. B.

[7] Fedotov A.K., Prischepa S.J., Fedotova J.A., Doctor V.G.B., Doctor A.A.R., Komissarov I.V., Kovalchuk N.G., Vorobyova S.A., Ivashkevich O.A. (2020). Electrical conductivity and magnetoresistance in twisted graphene electrochemically decorated with Co particles. Physica E.

[8] Zhang X.-G., Butler W.H. (1995). Conductivity of metallic films and multilayers. Phys. Rev. B.

[9] Levy P.M., Zhang S., Fert A. (1990). Electrical conductivity of magnetic multilayered structures. Phys. Rev. Lett..

[10] Lu J., Xu X., Greenblatt M., Jin R., Tinnemans P., Licciardello S., van Delft M.R., Buhot J., Chudzinski P., Hussey N.E. (2019). Emergence of a real-space symmetry axis in the magnetoresistance of the one-dimensional conductor *L**i*_0.9_*M**o*_6_*O*_17_. Sci. Adv..

[11] Mansour A.M. (2020). Magnetic sensors and geometrical magnetoresistance: A review. J. Met. Mater. Miner..

[12] Guimarães A.P. (2009). Principles of Nanomagnetism.

[13] Nickel J. (1995). Magnetoresistance Overview HP Labs Technical Reports HPL-95-60.

[14] Thomson W. (1857). On the electro-dynamic qualities of metals: Effects of magnetization on the electric conductivity of nickel and of iron. Proc. R. Soc. Lond..

[15] Groenendijk D.J., Manca N., de Bruijckere J., Mafalda A., Monteiro R.V.L., Gaudenzi R., Zant H.S.J.V., Caviglia A.D. (2020). Anisotropic magnetoresistance in spin–orbit semimetal *S**r**I**r**O*_3_. Eur. Phys. J. Plus.

[16] Bychkov Y.A., Rashba E.I. (1984). Properties of a 2D electron gas with lifted spectral degeneracy. Sov. Phys. JETP Lett..

[17] Dresselhaus G. (1955). Spin-orbit soupling effects in zinc blende structures. Phys. Rev..

[18] Su Y.-H., Chen S.-H., Hu C.D., Chang C.-R. (2016). Competition between spin-orbit interaction and exchange coupling within a honeycomb lattice ribbon. J. Phys. D Appl. Phys..

[19] Ganguly S., Maiti S.K., Sil S. (2021). Favorable thermoelectric performance in a Rashba spin-orbit coupled ac-driven graphene nanoribbon. Carbon.

[20] Maiti S.K. (2011). Determination of Rashba and Dresselhaus spin-orbit fields. J. Appl. Phys..

[21] Dey M., Maiti S.K., Karmakar S.N. (2012). Spin Hall effect in a Kagome lattice driven by Rashba spin-orbit interaction. J. Appl. Phys..

[22] Ganguly S., Basu S., Maiti S.K. (2018). Controlled engineering of spin-polarized transport properties in a zigzag graphene nanojunction. Europhys. Lett..

[23] Thompson S.M. (2008). The discovery, development and future of GMR: The Nobel Prize 2007. J. Phys. D Appl. Phys..

[24] Dagotto E. (2003). Brief Introduction to Giant Magnetoresistance (GMR). Nanoscale Phase Separation and Colossal Magnetoresistance.

[25] Núñez A.S., Duine R.A., Haney P., MacDonald A.H. (2006). Theory of spin torques and giant magnetoresistance in antiferromagnetic metals. Phys. Rev. B.

[26] Baibich M.N., Broto J.M., Fert A., Dau F.N.V., Petroff F., Etienne P., Creuzet G., Friederich A., Chazelas J. (1988). Giant Magnetoresistance of (001)Fe/(001)Cr Magnetic Superlattices. Phys. Rev. Lett..

[27] Weiss R., Mattheis R., Reiss G. (2013). Advanced giant magnetoresistance technology for measurement applications. Meas. Sci. Technol..

[28] Schmaus S., Bagrets A., Nahas Y., Yamada T.K., Bork A., Bowen M., Beaurepaire E., Evers F., Wulfhekel W. (2011). Giant magnetoresistance through a single molecule. Nat. Nanotech..

[29] Gerstner E. (2007). Nobel Prize 2007: Fert and Grünberg. Nat. Phys..

[30] Tian Y., Yan S.S. (2013). Giant magnetoresistance: History, development and beyond. Sci. China Phys. Mech. Astron..

[31] Mathon J. (1997). Tight-binding theory of tunneling giant magnetoresistance. Phys. Rev. B.

[32] Tsymbal E.Y., Mryasov O.N., LeClair P.R. (2003). Spin-dependent tunnelling in magnetic tunnel junctions. J. Phys. Condens. Matter.

[33] Inoue J., Maekawa S. (1996). Theory of tunneling magnetoresistance in granular magnetic films. Phys. Rev. B.

[34] Mathon J., Umerski A. (2001). Theory of tunneling magnetoresistance of an epitaxial *F**e*/*M**g**O*/*F**e*(001) junction. Phys. Rev. B.

[35] Ramirez A.P. (1997). Colossal magnetoresistance. J. Phys. Condens. Matter.

[36] Baldini M., Muramatsu T., Sherafati M., Mao H.-K., Malavasi L., Postorino P., Satpathy S., Struzhkin V.V. (2015). Origin of colossal magnetoresistance in *LaMn*0_3_ manganite. Proc. Natl. Acad. Sci. USA.

[37] Sen C., Alvarez G., Dagotto E. (2007). Competing Ferromagnetic and Charge-Ordered States in Models for Manganites: The Origin of the Colossal Magnetoresistance Effect. Phys. Rev. Lett..

[38] Sun J., Kosel J. (2013). Extraordinary Magnetoresistance in Semiconductor/Metal Hybrids: A Review. Materials.

[39] Hewett T.H., Kusmartsev F.V. (2010). Geometrically enhanced extraordinary magnetoresistance in semiconductor-metal hybrids. Phys. Rev. B.

[40] Pletikosić I., Ali M.N., Fedorov A.V., Cava R.J., Valla T. (2014). Electronic Structure Basis for the Extraordinary Magnetoresistance in *W**T**e*_2_. Phys. Rev. Lett..

[41] Ennen I., Kappe D., Rempel T., Glenske C., Hütten A. (2016). Giant Magnetoresistance: Basic Concepts, Microstructure, Magnetic Interactions and Applications. Sensors.

[42] Camblong H.E. (1995). Linear transport theory of magnetoconductance in metallic multilayers: A real-space approach. Phys. Rev. B.

[43] Gijs M.A.M., Bauer G.E.W. (2006). Perpendicular giant magnetoresistance of magnetic multilayers. Adv. Phys..

[44] Tsymbal E.Y., Pettifor D.G. (2001). Perspective of giant magnetoresistance. Solid State Phys..

[45] Xiong P., Xiao G., Wang J.Q., Xiao J.Q., Jiang J.S., Chien C.L. (1992). Extraordinary Hall effect and giant magnetoresistance in the granular Co-Ag system. Phys. Rev. Lett..

[46] Parkin S.S.P. (1995). Giant magnetoresistance in magnetic nanostructures. Annu. Rev. Mater. Sci..

[47] Banik S., Das K., Paramanik T., Lalla N.P., Satpati B., Pradhan K., Das I. (2018). Huge magnetoresistance and ultrasharp metamagnetic transition in polycrystalline *S**m*_0.5_*C**a*_0.25_*S**r*_0.25_*M**n**O*_3_. NPG Asia Mater..

[48] Patra M., Maiti S.K., Sil S. (2019). Engineering magnetoresistance: A new perspective. J. Phys. Condens. Matter.

[49] Piraux L., George J.M., Despres J.F., Leroy C., Ferain E., Legras R. (1994). Giant magnetoresistance in magnetic multilayered nanowires. Appl. Phys. Lett..

[50] Li J., Cheng C., Paiva T., Lin H.-Q., Mondaini R. (2018). Giant Magnetoresistance in Hubbard Chains. Phys. Rev. Lett..

[51] Poulsen K., Zinner N.T. (2021). Giant Magnetoresistance in Boundary-Driven Spin Chains. Phys. Rev. Lett..

[52] Marti X., Fina I., Frontera C., Liu J., Wadley P., He Q., Paull R.J., Clarkson J.D., Kudrnovský J., Turek I. (2014). Room-temperature antiferromagnetic memory resistor. Nat. Mater..

[53] Bhatti S., Sbiaa R., Hirohata A., Ohno H., Fukami S., Piramanayagam S.N. (2017). Spintronics based random access memory: A review. Mater. Today.

[54] Raffel J., Crowder T. (1964). A Proposal for an Associative Memory Using Magnetic Films. IEEE Trans. Electron. Comp..

[55] Daughton M., Pohm A.V., Fayfield R.T., Smith C.H. (1999). Applications of spin dependent transport materials. J. Phys. D Appl. Phys..

[56] Mott N.F. (1936). The electrical conductivity of transition metals. Proc. R. Soc. A.

[57] Gong G.Q., Gupta A., Xiao G., Qian W., Dravid V.P. (1997). Magnetoresistance and magnetic properties of epitaxial magnetite thin films. Phys. Rev. B.

[58] Martin L.W., Chu Y.H., Ramesh R. (2010). Advances in the growth and characterization of magnetic, ferroelectric, and multiferroic oxide thin flims. Mater. Sci. Eng. R.

[59] Bass J., Pratt W.P. (2007). Spin-diffusion lengths in metals and alloys, and spin-flipping at metal/metal interfaces: An experimentalist’s critical review. J. Phys. Condens. Matter.

[60] Sarkar M., Dey M., Maiti S.K., Sil S. (2020). Engineering spin polarization in a driven multistranded magnetic quantum netword. Phys. Rev. B.

[61] Koley A., Maiti S.K., Silva J.H.O., Laroze D. (2021). Spin Dependent Transport through Driven Magnetic System with Aubry-Andre-Harper Modulation. Appl. Sci..

[62] H S.A.A., André G. (1980). Analyticity breaking and Anderson localization in incommensurate lattices. Ann. Isr. Phys. Soc..

[63] Harper P.G. (1955). Single Band Motion of Conduction Electrons in a Uniform Magnetic Field. Proc. Phys. Soc. Lond. Sect. A.

[64] Hofstadter D.R. (1976). Energy levels and wave functions of Bloch electrons in rational and irrational magnetic Telds. Phys. Rev. B.

[65] Sil S., Maiti S.K., Chakrabarti A. (2008). Metal-insulator transition in an aperiodic ladder network: An exact result. Phys. Rev. Lett..

[66] Roy N., Sharma A. (2019). Study of counterintuitive transport properties in the Aubry-André-Harper model via entanglement entropy and persistent current. Phys. Rev. B.

[67] Ganeshan S., Pixley J.H., Sarma S.D. (2015). Nearest Neighbor Tight Binding Models with an Exact Mobility Edge in One Dimension. Phys. Rev. Lett..

[68] Kraus Y.E., Lahini Y., Ringel Z., Verbin M., Zilberberg O. (2012). Topological States and Adiabatic Pumping in Quasicrystals. Phys. Rev. Lett..

[69] Lahini Y., Pugatch R., Pozzi F., Sorel M., Morandotti R., Davidson N., Silberberg Y. (2009). Observation of a localization transition in quasiperiodic photonic lattices. Phys. Rev. Lett..

[70] Roy S., Maiti S.K. (2019). Tight-binding quantum network with cosine modulations: Electronic localization and delocalization. Eur. Phys. J. B.

[71] Patra M., Maiti S.K. (2017). Externally controlled high degree of spin polarization and spin inversion in a conducting junction: Two new approaches. Sci. Rep..

[72] Patra M., Maiti S.K. (2019). Controlled charge and spin current rectifications in a spin polarized device. J. Magn. Magn. Mater..

[73] Dey S., Daw D., Maiti S.K. (2020). Flux driven circular current and near zero field magnetic response in an Aubry ring: High-to-low conducting switching action. Europhys. Lett..

[74] Delplace P., Gómez-Lexoxn A., Platero G. (2013). Merging of Dirac points and Floquet topological transitions in ac-driven graphene. Phys. Rev. B.

[75] Gómez-Lexoxn A., Platero G. (2013). Floquet-Bloch Theory and Topology in Periodically Driven Lattices. Phys. Rev. Lett..

[76] Asobóth J.K., Tarasinski B., Delplace P. (2014). Chiral symmetry and bulk-boundary correspondance in periodically driven one-dimensional systems. Phys. Rev. B.

[77] Runder M.S., Linder N.H. (2020). Band structure engineering and non-equilibrium dynamics in Floquet topological insulators. Nat. Rev. Phys..

[78] Eckardt A., Anisimovas E. (2015). High-frequency approximation for periodically driven quantum systems from a Floquet-space perspective. New J. Phys..

[79] Liang P., Marthaler M., Guo L. (2018). Floquet many-body engineering: Topology and many-body physics in phase space lattices. New J. Phys..

[80] Datta S. (1995). Electronic Transport in Mesoscopic Systems.

[81] Wu B.H., Cao J.C. (2008). A Floquet-Green’s function approach to mesoscopic transport under ac bias. J. Phys. Condens. Matter.

[82] Zhang X.W., Liu Y.L. (2019). Electronic transport and spatial current patterns of 2D electronic system: A recursive Grenn’s function method study. AIP Adv..

[83] Peres N., Stauber T., Santos J.M.B.L.D. (2009). Lattice Green’s function approach to the solution of the spectrum of an array of quantum dots and its linear conductance. Phys. Rev. B.

[84] Bi M.X., Yan X.H., Xiao Y., Guo H. (2019). Time-dependent Green’s function approach to spin transport assisted by nonclassical light. Phys. Rev. B.

[85] Fisher D.S., Lee P.A. (1981). Relation between conductivity and transmission matrix. Phys. Rev. B.

[86] Karuppasamy P., Kamalesh T., Anitha K., Kalam S.A., Pandian M.S., Ramasamy P., Verma S., Rao S.V. (2018). Synthesis, crystal growth, structure and characterization of a novel third order nonlinear optical organic single crystal: 2-amino 4, 6-dimethyl pyrimidine 4-nitrophenol. Opt. Mater..

[87] Murugesan M., Paulraj R., Perumalsamy R., Kumar M.K. (2020). Growth, photoluminescence, lifetime, and laser damage threshold studies of 1, 3, 5-triphenylbenzene (TPB) single crystal for scintillation application. Appl. Phys. A.

